# Design and realization of a novel haptic graspable interface for augmenting touch sensations

**DOI:** 10.3389/frobt.2022.927660

**Published:** 2022-09-28

**Authors:** Vijay Kumar Pediredla, Karthik Chandrasekaran, Srikar Annamraju, Asokan Thondiyath

**Affiliations:** ^1^ AI and Robotics Technology PARK, Indian Institute of Science, Bangalore, India; ^2^ School of Interdisciplinary Design and Innovation, Indian Institute of Information Technology, Design and Manufacturing, Chennai, India; ^3^ Health Care Engineering Systems Center, Coordinated Science Laboratory, University of Illinois at Urbana Champaign, Champaign, IL, United States; ^4^ Robotics Lab, Department of Engineering Design, Indian Institute of Technology Madras, Chennai, India

**Keywords:** haptic interfaces and devices, kinesthetic feedback, mechanism design and analysis, tactile feedback, vibroactuation, virtual/augmented reality

## Abstract

A novel haptic grasper that renders touch sensations to the user in 3-DoF (degrees of freedom), namely linear, rotary, and grasping motions, is presented. The touch sensations of the grasper include the combination of kinesthetic and tactile modalities such as stiffness, texture, and shape. The device is equipped with two swappable modular segments that provide stiffness and shape sensations. To increase the haptic fidelity, the textural surfaces that surround the outer surface of the segments are equipped with vibro-actuators underneath them. These vibro-actuators contribute to increasing the number of perceivable textures by varying amplitude, frequency, duration, and envelope of vibrations. The proposed device is characterized in terms of stiffness, shape and texture rendering capabilities. The experimental results validate the effectiveness of the developed haptic grasper in virtual/remote interactions. Also, the user studies and statistical analysis demonstrate that the users could perceive the high-fidelity haptic feedback with the unified sensations of kinesthetic and tactile cues.

## Introduction

Human-machine interfaces (HMIs) provide intuitive graphical and manipulation capabilities that assist the user in interacting with machines (robots or virtual environments). The conventional HMIs, such as a joystick, keyboard, smartphones, etc., have been replaced by advanced interfaces such as virtual reality (VR) and augmented reality (AR) haptic devices that render compelling and natural sensations. Haptic devices play an essential role in immersing the user in teleoperation and virtual/augmented environments by providing force/touch information. In recent years, these devices had significant attention and have been actively pursued by many research groups worldwide. The bifacial feature of these interfaces guarantees safe/precise sensing and manipulation, unlike the existing unidirectional interfaces that are limited to visual, vibratory, or auditory feedback. Additionally, introducing haptic interfaces into the human-machine systems elevates the interactions to higher dimensions by conveying physical features through kinesthetic and tactile stimuli (Culbertson et al., 2018). Kinesthetic stimuli provide information related to applied forces and movements through muscle and joint receptors. On the other hand, the tactile stimuli offer a sense of local physical properties through mechanoreceptors in the skin. Kinesthetic devices provide important features such as wide dynamic range, high forces, and many degrees of freedom (DoF) (Pacchierotti et al., 2017). These features greatly support rendering a realistic touch. However, the bulkiness and expensiveness confine its usage in various applications. Tactile devices stimulate skin through actuators and provide cues such as shape, slip, and texture. Most of these devices are portable/wearable and inexpensive. However, providing convincing sensations (both cutaneous and kinesthetic feedback) is not possible with such devices. This limits the use of such devices in performing tasks related to manipulation, teleoperated surgery, etc. (Lim et al., 2015). Many practical applications require accurate spatial recognition to recognize the local shape and texture tangibly, which may not be provided by the existing tactile feedback devices. Furthermore, generating tactile cues is challenging as it necessitates miniaturization along with high resolution and high frequency. In general, the current feedback devices implement kinesthetic and tactile feedback stimuli separately. Researchers have shown that kinesthetic or tactile stimulation alone cannot impact the user interactions, as the rendering of each interaction depends on its combination (Scilingo et al., 2010; Fani et al., 2018). Therefore, providing the synergistic combination of these two stimuli is necessary for making the interactions appealing and realistic.

Haptic interfaces are categorized into grounded devices (tool-based and skeleton-frame) and ungrounded devices such as exoskeleton or wearable devices (Pacchierotti et al., 2017). Mid-range haptic devices such as Phantom Touch™ (3D systems) and Omega.3™ (Force dimension) use a stylus/tool surrogate for a single point interaction (Jang and Lee, 2014), thus reducing the overall natural perception while identifying and manipulating virtual objects. Also, the single-contact point interfaces largely constrain the user in acquiring the desired sensations. In contrast, the multi-point contact interfaces offer a higher haptic fidelity by increasing the perceptual response range (Galiana and Ferre, 2013). Therefore, researchers have developed multi-fingered haptic devices to enhance perception, manipulation, and grasping power (Bergamasco et al., 2007). Skeleton-frame devices proposed in the literature are Hiro III (Endo et al., 2009), a five-fingered interface that provides the size and weight of the virtual object, and SPIDAR (Murayama et al., 2004), a bimanual device that generates the force feedback in 6-DoF. However, Hiro III has limited rendered workspace and gestures, and SPIDAR has limited force bandwidth. Exoskeleton devices help the applications requiring a large extent of maneuver. In (Frisoli et al., 2007), a two-point contact model that reproduces forces in 3-DoF was proposed. CyberGrasp™ (CyberGlove systems) and Wolverine (Choi et al., 2016) are used for consumer applications because of their low weight, low cost, and large motion range. However, the user’s experience with most interfaces is non-intuitive and unnatural due to design complexities, constraints on inertia and interfacing methods, back drivability, and actuator power (Najdovski et al., 2014). Moreover, the lack of tactile feedback in these interfaces limits the transparency. The wearable haptic interfaces provide cutaneous cues through pin-arrays, vibrations, and contact forces. In (Schorr and Okamura, 2017; Lee et al., 2019), a 3-DoF device is developed to convey lateral and normal forces of virtual objects. In (Ohka et al., 2005), large pressure strokes are provided by varying the pin matrix heights. However, the display resolution is limited because of the actuators’ spatial density. A vibrotactile display (Asano et al., 2015) is used to vary the roughness and texture. However, the tactile fidelity is subjugated by intense vibrations when the rendered texture changes. In (Whitmire et al., 2018), a haptic revolver with interchangeable wheels comprising textures and shapes is proposed. However, rendering stiffness and quick access to textures remains a challenge. Most tactile devices do not include stiffness, an essential haptic modality. Some state-of-the-art tactile devices can generate combinations of stiffness with texture (Benko et al., 2016) and stiffness with size and shape (Sun et al., 2018) but cannot provide either shape or texture.

Additionally, the workspace, number of rendered textures, and resolution for these devices are limited. Nonetheless, these interfaces may not provide faithful haptic sensations because of the lack of a proper combination of haptic stimuli and grasping motion. In (Lederman and Klatzky, 1997), it is determined that integrating stiffness with texture could provide an accurate feel of perceptual cues because of the influence of material properties over structural properties. Hence, developing an adaptable graspable interface rendering both kinesthetic and tactile feedback is needed.

In this paper, we present the design of a graspable haptic interface that provides unified kinesthetic and tactile feedback by rendering the stiffness, texture, and shape modalities in 3-DoF. The goal here is to enhance transparency while interacting with diverse environments. The proposed device includes perception/grasping abilities through multi-contact sensations and has a modular configuration consisting of replaceable modules for providing various sensations. The device also uses a novel flexure-based backlash-compensated mechanism for power transmission to improve haptic fidelity. One of the critical contributions is in emulating the textural feedback by using an arrangement of passive textures backed by vibrotactile actuators. Other contributions are in the grasper’s design with zero backlash, low friction, low inertia, high force bandwidth, and high back drivability.

## Design and working principle

**FIGURE 1:**
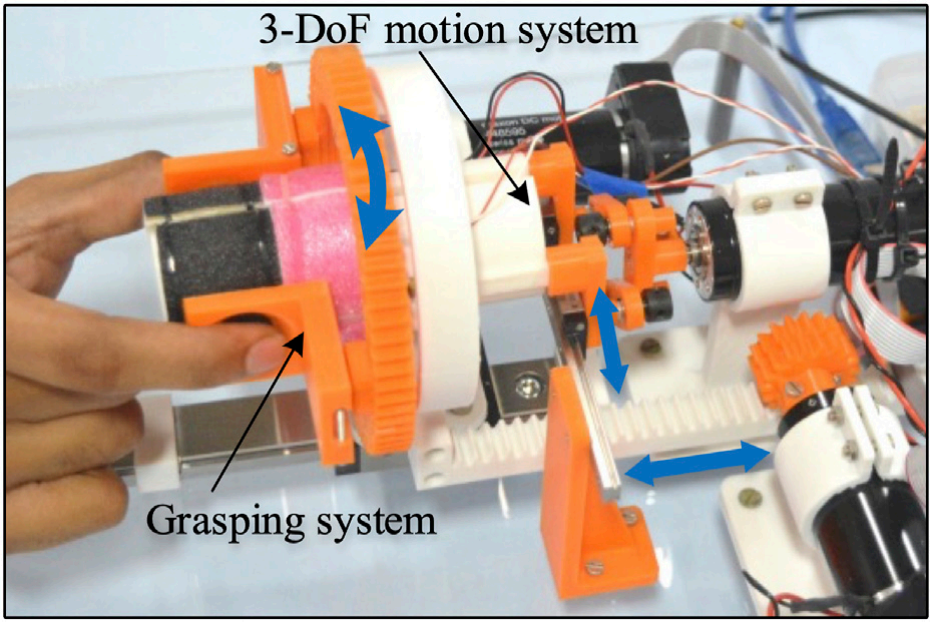
The proposed haptic grasper with grasping and 3-DoF motion subsystems uses segment modules to provide kinesthetic and tactile feedback. The segment modules are interchangeable and can be varied to render a variety of shapes, textures, edges, and active components corresponding to remote/virtual environments.

The haptic device has been designed to provide 3-DoF motion with unified kinesthetic and tactile feedback by rendering the stiffness, texture, and shape modalities. The goal of developing this haptic grasper is to enhance transparency while interacting with diverse environments. Figure 1 shows the prototype device along with its subsystems. It has two major subsystems: 1) Grasping system along with texture rendering and 2) 3-DoF motion mechanism.

The grasping system allows users to interact with a virtual environment using their fingers. This system comprises a grasper with texture rendering modules. The vibro-actuators placed beneath the texture module provide the perception of textures by varying the vibration characteristics. The grasper can move in 3-DoF (linear, rotary, and grasping motions) provided by the motion mechanism, actuated using three motors (DC Maxon motors: RE-30). Each of the motors is controlled using Maxon EPOS 4 50/5 CAN controller. The design details of these modules are explained in the following sections.

### Grasper system

**FIGURE 2:**
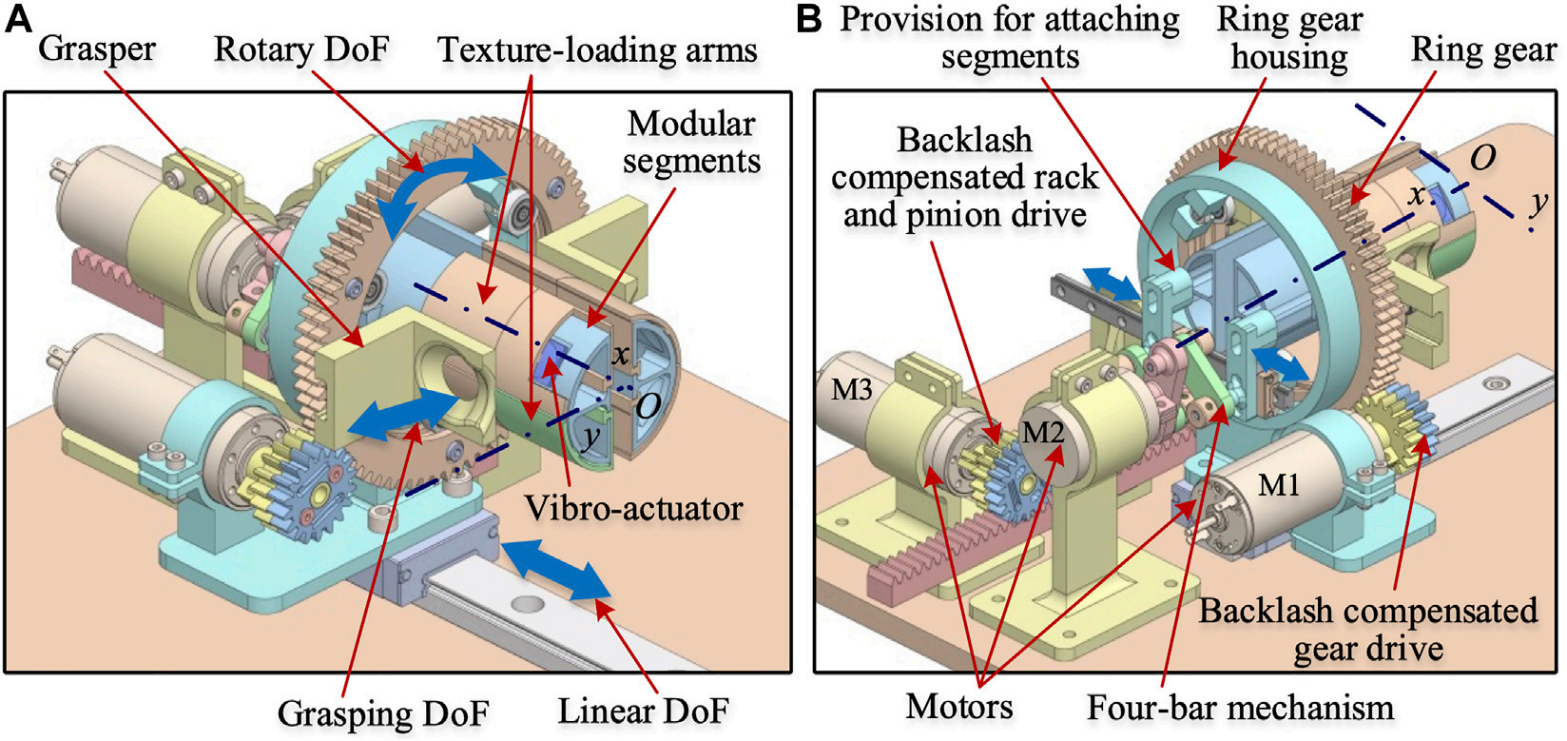
CAD model of the proposed haptic grasper **(A)** Isometric view (partial section of the textural loading arm is hidden to reveal vibro-actuator) **(B)** Another view of the grasper showing the motors and drive mechanism. The double-headed arrows in each model show the grasper’s direction of motion. The modular segments are attached with texture loading arms and vibro-actuators, which are fixed to a 4-bar mechanism and can only move in a lateral direction providing the grasping DoF.

The grasping system consists of a pair of passive graspers that allow the user to sense and explore the virtual/remote environment using the fingers in 3-DoF, namely the grasping motion (radially inwards), linear motion (along the *x*-axis), and rotary motion (about the *x*-axis), as shown in Figure 2. The grasping DoF allows the user to sense the stiffness and shape information, and the linear and rotary DoF renders shape and textural information of the virtual/remote environment. The graspers can move around the texture-loading arms and have an opening that allows the user’s fingertips to contact the textural surfaces. There are two semi-circular modular segments at the centre of the grasper mechanism. These modular segments are fixed to a 4-bar mechanism, as shown in Figure 2B, and can only be moved laterally, thus providing the grasping DoF. These segments can be replaceable with another set of segments of different geometries that can comprise edges, shapes, and active components corresponding to virtual environments. These two segments have a relative motion along the *y*-axis. These segments are divided into multiple sections, and textures/shapes of various features are attached to these sections through the texture-loading arms, as shown in Figure 2.

An array of vibro-actuators is placed between the texture loading arms and the modular segments, and are configured to emulate multiple textures by varying the vibrational characteristics. Texture-loading arms provide the preload, and they are affixed with multiple passive surfaces having fine to coarse textures. The combination of vibro-actuators and passive textures enables realizing a multitude of surfaces with varying tactile properties. The segments and textures can be swapped to represent virtual/remote environments with different shapes and textural properties.

### 3-DoF motion mechanism

**FIGURE 3:**
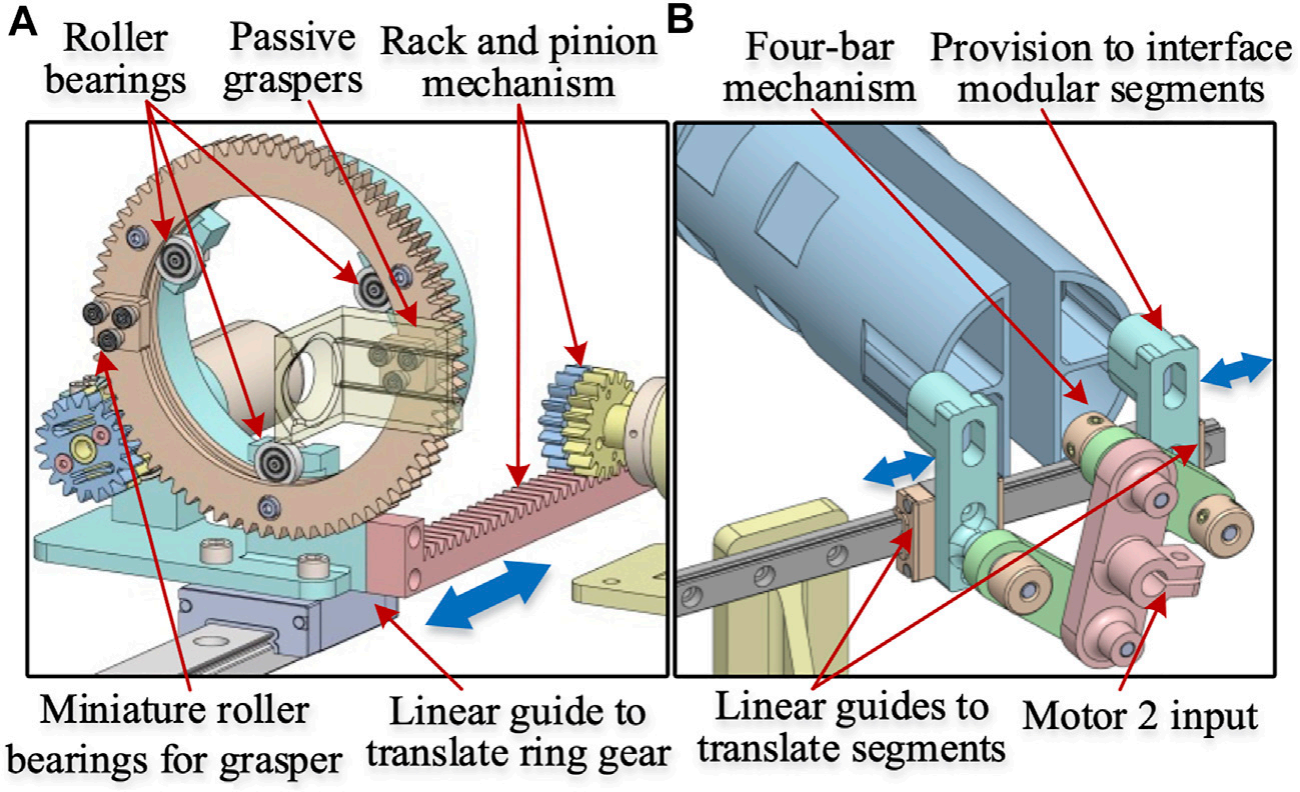
Subassemblies of the haptic grasper **(A)** Ring gear mechanism **(B)** Four-bar mechanism for moving modular segments. The ring gear mechanism holds the passive graspers through miniature roller bearings that allow the lateral movement of the graspers (grasping DoF). Also, the other roller bearings allow the rotary DoF. The 4-bar mechanism is attached to the modular segments through a provision component that moves along with the passive graspers providing the grasping DoF.

The mechanisms used for getting the 3-DoF motion are shown in Figures 2, 3. The grasper’s rotational motion is obtained using a ring gear driven by a motor M1. The two passive graspers are connected to the ring gear by bearings that allow the graspers to move radially, as shown in Figure 3A. The ring gear is attached to the housing by three roller bearings, which provide rotary DoF to the graspers. A low-friction linear guide holds the ring gear housing and is driven by a backlash compensated gear drive, as shown in Figure 3A.

The individual modular segments are attached to the linear guide (through provisions) that move along the *y*-axis and are driven by motor M2 through a 4-bar mechanism, as shown in Figure 3B. The linear motion of the grasper in the *x*-direction is obtained through a backlash compensated rack and pinion mechanism driven by motor M3, as shown in Figure 2B. Further details of these mechanisms are explained in the following sections.

### Flexure based backlash compensated gear drive

**FIGURE 4:**
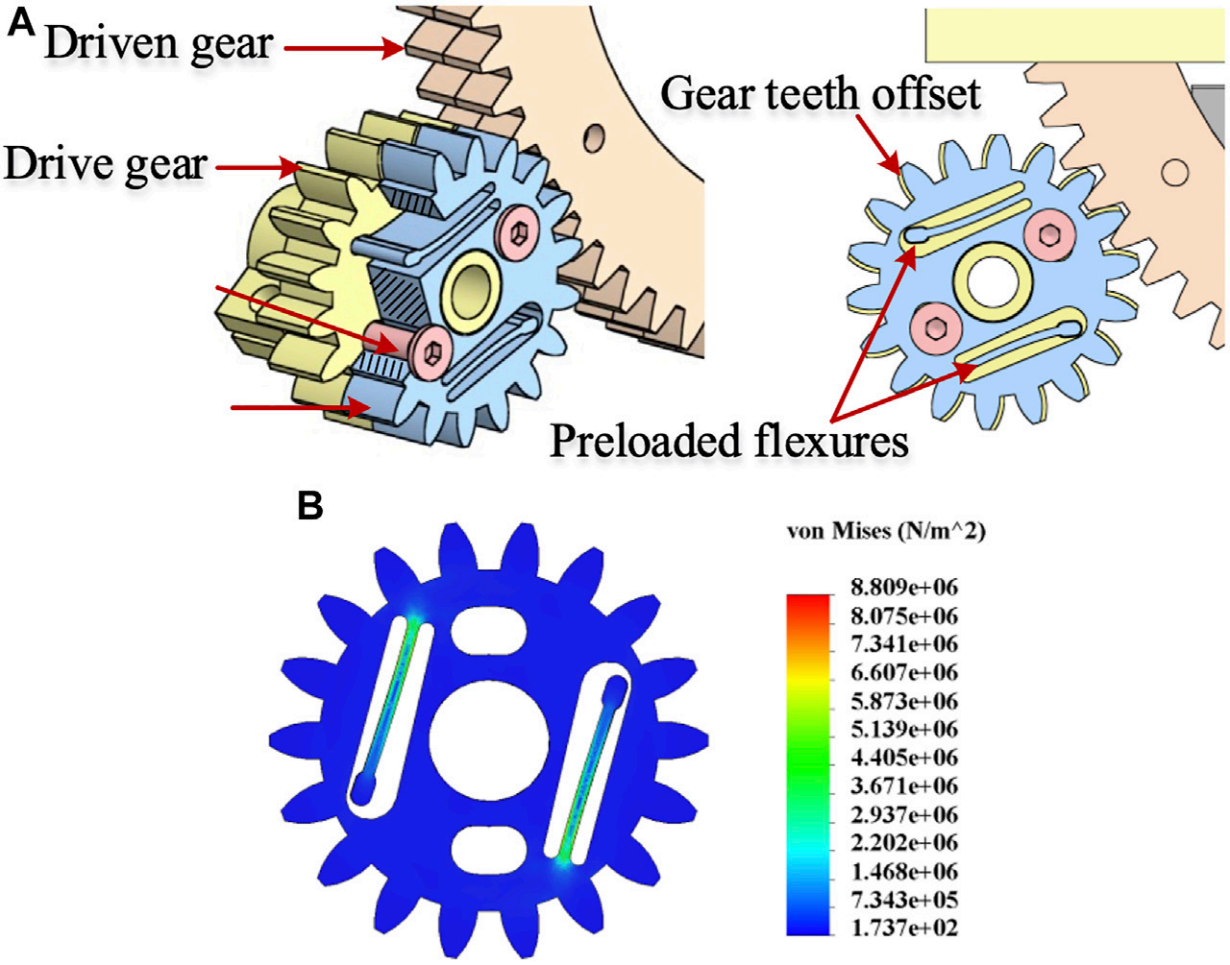
**(A)** Flexure based implementation of backlash compensated gear drive **(B)** FEA analysis of backlash compensating flexures. The flexure behaves as a serial elastic element in the gear drive, leading to an asymmetric torsional stiffness of the gear drive when reversing motion. The preloaded flexure negates the backlash and leads to an equal torsional stiffness of the gear drive in both directions (clockwise and anti-clockwise).

Gear-driven transmission, compared to cable transmission, offers high stiffness but is prone to backlash. An anti-backlash mechanism, typically used in literature, consists of two drive gears mounted co-axially to a common shaft to remove circumferential backlash. One of the gears is rigidly mounted to the drive shaft, while the other is connected through a pair of springs and is floating. The springs are preloaded, and during mounting, the floating gear negates any backlash due to the springs’ torque. Since one of the gears is floating and the spring behaves like a serial elastic element, the system exhibits limited stiffness in one of the directions of gear motion. This stiffness limitation can be addressed by locking both gears. We propose a flexure-based backlash compensated gear drive, as shown in Figure 4A. The proposed anti-backlash mechanism uses a pair of flexures to preload the split gear arrangement. The floating gear has a pair of flexures with a protrusion at the flexure end that engages holes in the other gear. The holes are located such that during assembly, the floating gear must be rotated to facilitate the engagement of the mating gear teeth. The flexures bend during assembly and exert torque on the drive gears. Thus, the floating gear rotates due to flexure’s torque and negates any backlash during initial assembly. After assembly, a pair of clamping screws are tightened to clamp the two gears, thus becoming an integral component. As a result, the flexures do not carry any driving torque during operation. Therefore, this arrangement provides good stiffness in either of the rotating directions, and there is no issue of springs’ influencing the transmission stiffness. Also, as the flexures are an integral part of the drive gear, the mechanical complexity of transmission is greatly simplified. The function of the flexure based spring is to facilitate automatic backlash compensation in the gear drive. The same concept has been utilized for driving the rack and pinion mechanism. It is to be noted that a small clearance between the gear teeth is provided in practice for smooth operation in the conventional gear transmission. But this clearance invariably leads to the backlash in the gear drive. In our implementation, there is clearance between the mating gear pairs. But this clearance is negated by the flexural spring. Additionally, there are no cogging or noise effects because choosing the involute profile for gear teeth strictly follows the gearing law, which ensures constant angular velocity for the output gear. Also, the minimum no. of teeth for the pinion gear of the motors is kept above 17 to prevent tooth interference.

A Finite Element Analysis (FEA) on the flexure has been done to estimate bending stresses, and the results are shown in Figure 4B. The stresses are well within the allowable limit for the 3D printed prototype material - ABS (flexural strength—70.5 MPa).

### Four-bar mechanism

**FIGURE 5:**
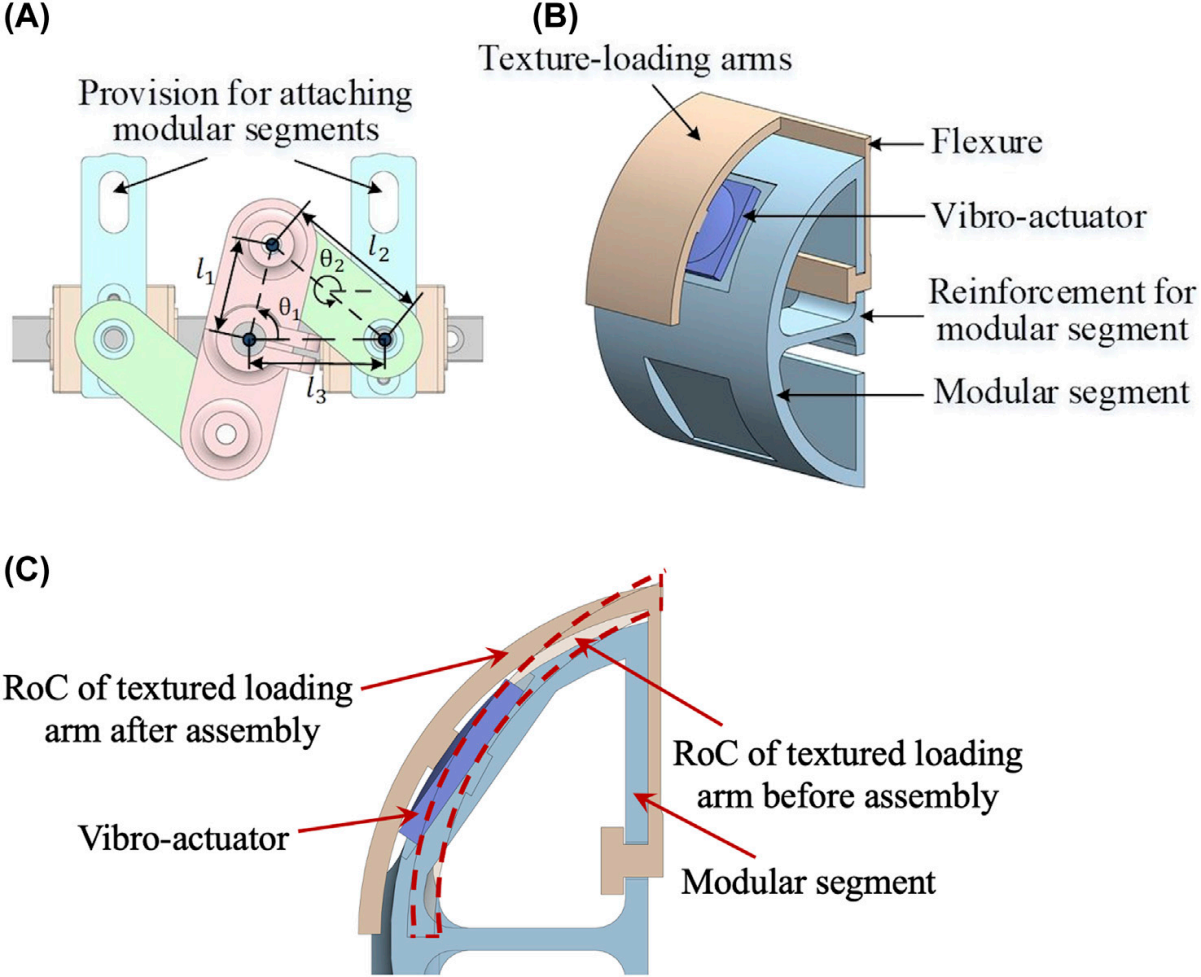
**(A)** Four-bar mechanism **(B)** Partially sectioned view of the modular segment with texture-loading arms and vibro-actuator **(C)** Vibro-actuator preloading. Four-bar mechanism renders stiffness to the user through the lateral movement of segments. The segments are modular and can be swappable with various segments. The textural arm is 3D printed with a smaller radius of curvature (roc), and after the assembly, the roc increases to accommodate the vibro-actuator leading to its preloading.

A separate motor controls the grasping stiffness of the device. A 4-bar slider-crank mechanism, as shown in Figure 5A, converts the rotary motion to the lateral grasping motion. The interchangeable modular segments can be plugged into the sliders using the provisions shown in Figure 5. Therefore, the stiffness of the graspers can be varied by controlling the motor’s torque. Additionally, the shape can be rendered by position control of the segments. The relation between the change in linear displacement ( x ) of segments and the rotary motion ( $\theta_{1}$ ) of the motor is given by

$$x=2\;\left(l_{1}\cos\left(\theta_{1}\right)+l_{2}\cos\left(\theta_{2}\right)-l_{3}\right)$$ (1)

$$\theta_{2}=\sin^{-1}\left(-\frac{l_{1}\sin\left(\theta_{1}\right)}{l_{2}}\right)$$ (2)

where $l_{1}=15\;mm$ ; $l_{2}=22.6\;mm$ and $l_{3}=29\;mm$ .

### Piezo haptic actuator

There are vibro-actuators (PowerHap-11G™, TDK) integrated into the modular segments to increase the textural sensations, thus recreating different variants of the attached textures. These actuators are mounted between the texture loading arms and modular segments. For optimal performance, a preload of around 7 N is applied (as per the manufacturer) to compress the actuators using the texture loading arm, as shown in Figure 5B. The texture loading arm is designed to open the circular part to facilitate the assembly. The flexure on the texture loading arm is flexed after assembly to provide the preload for the actuator, as shown in Figure 5B. The textural arm is 3D printed with a smaller radius of curvature (RoC), and after the assembly, the RoC increases to accommodate the vibro-actuator leading to its preloading, as shown in Figure 5C.

An I2C communication-based haptic driver (Piezo haptic flex module, Fyber Labs Inc.) controls the actuator’s vibrational characteristics. It is connected to an Arduino Uno controller, through which diverse waveforms are generated by varying amplitude, frequency, duration, and envelope of the vibrations. The waveform’s maximum frequency and quick response time of 500 Hz and 2 ms, respectively, enables the actuator to suit various haptics applications (Asano et al., 2015). Complex haptic waveforms can also be constructed and fed as an input to reconstruct a complicated textural surface. The vibro-actuator also functions as an integrated sensor capable of measuring applied forces up to 15 N at the end-effector. The user’s force applied to the integrated sensor acts as an input, generating the corresponding voltage. Also, the sensor is calibrated using standard weights to ensure accurate force measurement.

For better understanding of the working of the proposed haptic grasper some more figures are provided in the Supplementary Material.

## System characterization and transparency analysis

Experiments have been performed to determine the device characteristics and evaluate the haptic grasper’s performance for transparency and quality of rendering.

### Backlash analysis

**FIGURE 6:**
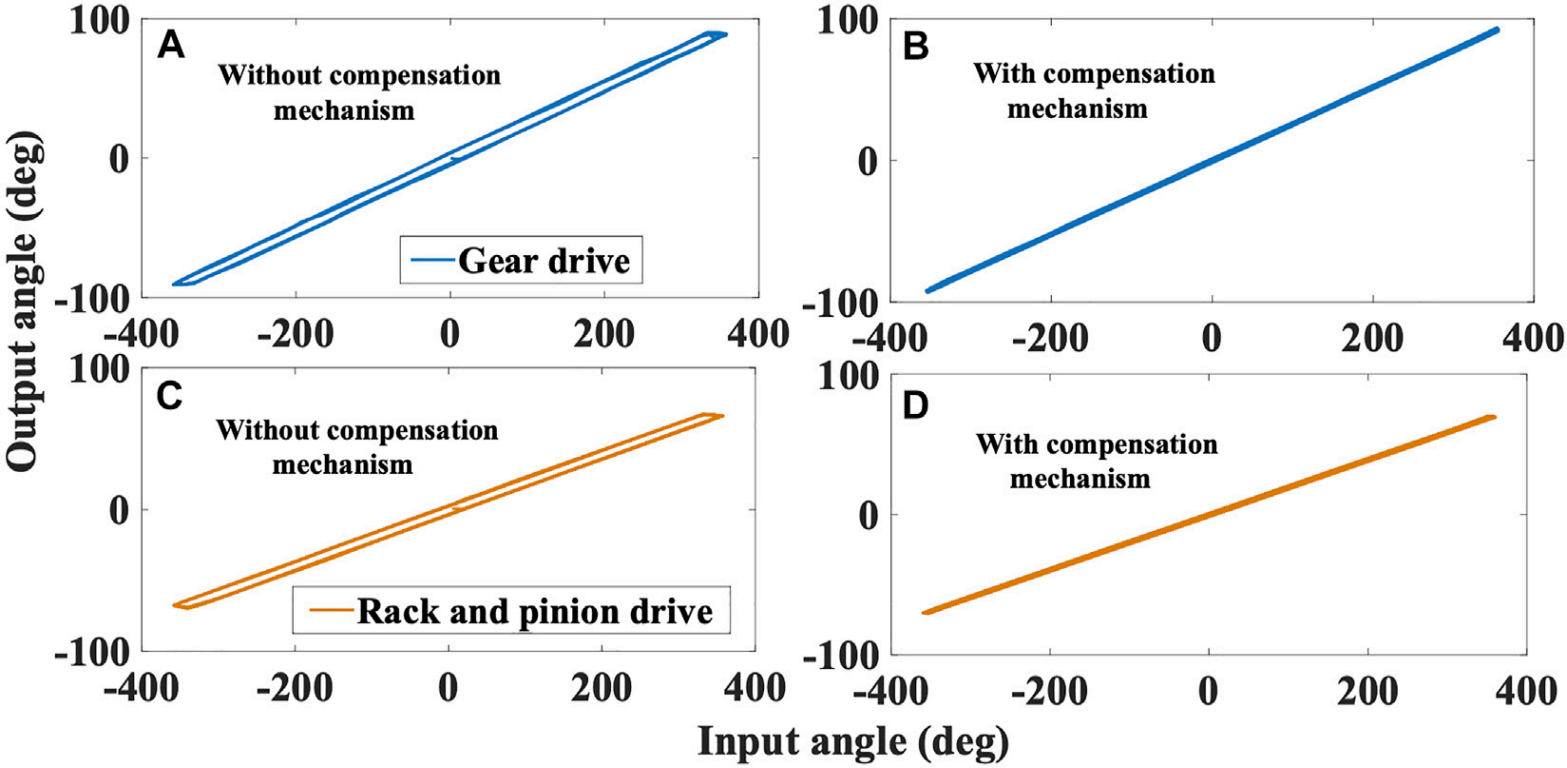
Backlash measurement with input-output hysteresis phenomenon for gear drive, and rack and pinion drive with/without compensation mechanism. **(A)** Gear drive without compensation **(B)** Gear drive with compensation **(C)** Rack and pinion drive without compensation **(D)** Rack and pinion drive with compensation.

To analyze the transparency in the haptic rendering of the device in 2-DoF (rotary and linear motion), the proposed flexure-based backlash compensated drives were considered. The input and output angles of the gear drive and rack and pinion drive were recorded to study the backlash of the device in 2-DoF. The gears attached to motors (M1 and M3) were given a complete rotation in the clockwise and counter-clockwise directions. The corresponding movement of the ring gear and rack and pinion was measured to compute the backlash. The angular rotation of M1 and M3 were measured using the motor’s integrated encoder. Similarly, the rotary motion of the ring gear and linear motion of the rack and pinion drive was measured using a calibrated camera. The camera data is processed through the Lucas-Kanade point tracker algorithm that utilizes the dominant points of the grasper and calculates the position/velocity from each video sequence frame. The hysteresis exhibited by the gear train for the rotary motion and the rack and pinion drive for linear motion of grasper assembly was computed with and without backlash compensation mechanism, as shown in Figure 6. The average backlash for both drives with and without compensation mechanism was 0.23 and 8.72 deg, respectively. The results show a significant decrease (about 97%) in backlash with the compensation mechanism, and thus the device provides transparent haptic sensations with the proposed gear and rack and pinion drives.

### Force rendering

**FIGURE 7:**
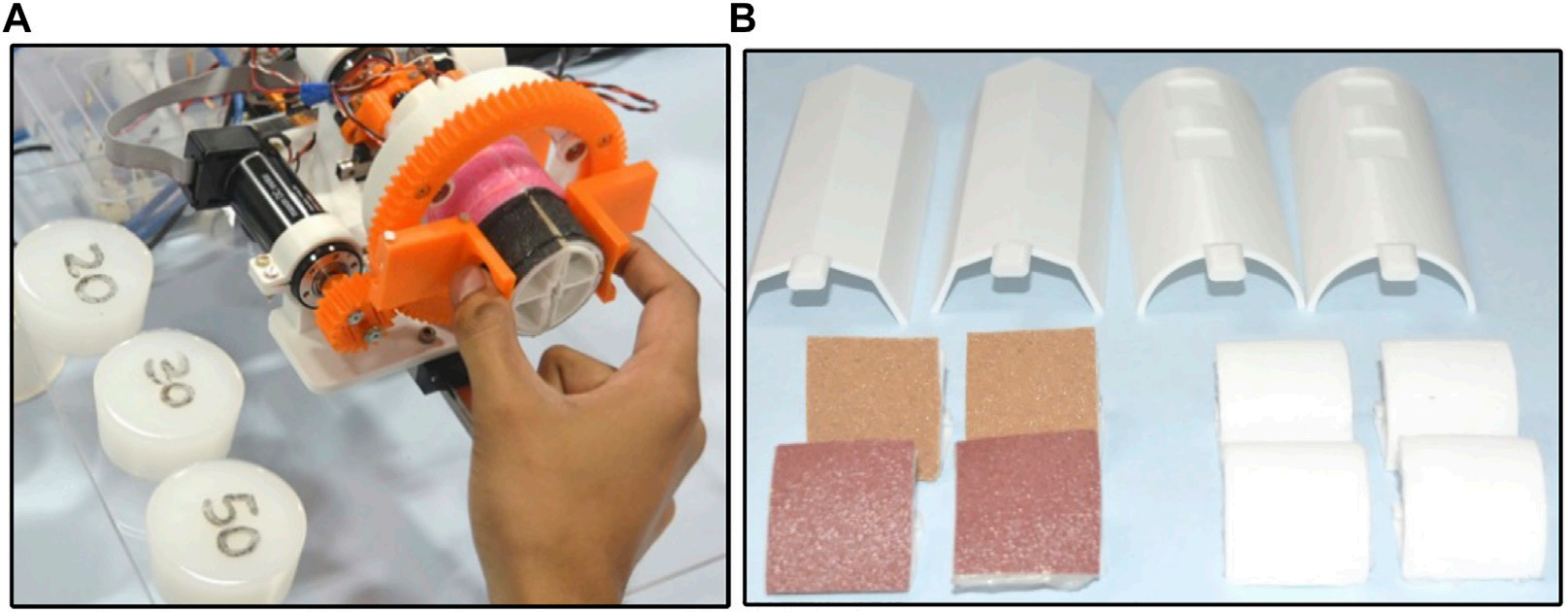
**(A)** Silicone specimens with Haptic grasper **(B)** Modular segments and textural-loading arms of the haptic grasper. The textural loading arms are equipped with a variety of textures, for instance, soft fabrics, smooth and rough surfaces, and so on. The modular segments allow customization based on the application.

**FIGURE 8:**
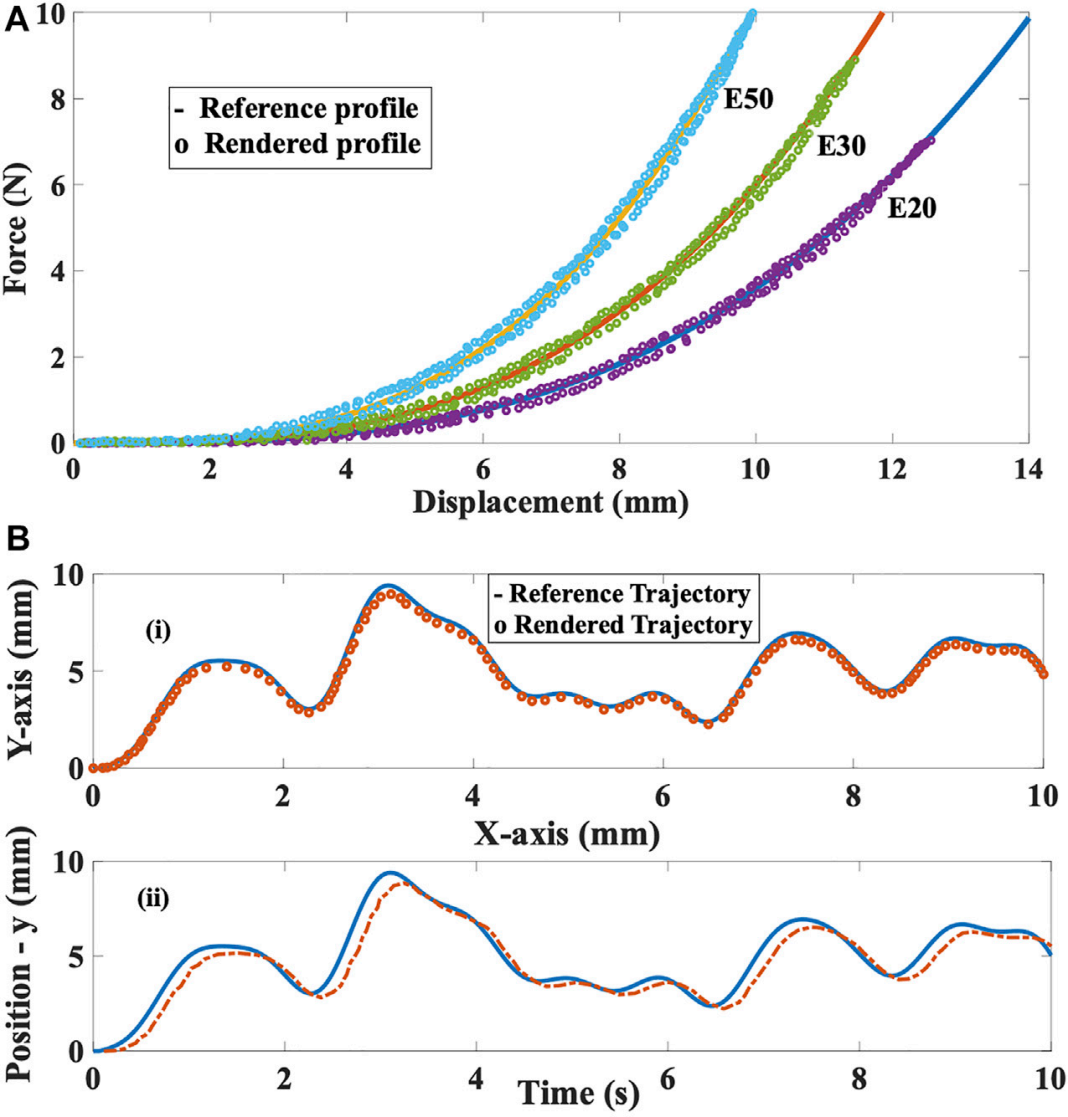
**(A)** Nonlinear stiffness tracking for E20, E30 and E50 **(B)** Shape profile comparison and position tracking in time domain along XY-plane.

To analyze the force rendering capability of the device, three workpieces of various stiffness were fabricated using silicone material (Ecoflex™–E20, E30, E50). The length and radius of the cylindrical workpieces were 3 and 6 cm, respectively, as shown in Figure 7A. Using a UTM (universal testing machine), these samples were tested to obtain force-displacement curves. The curves were approximated to the cubic polynomials using polynomial fitting in MATLAB and considered reference profiles, as shown in Figure 8A. The force-displacement characteristics for the three samples were given by

$$F\left(x\right)=k_{p}\left(x^{3}\right)$$ (3)

where $k_{p}$ (cubic stiffness coefficient ( $N/mm^{3}$ )) for E20, E30 and E50 are $3.6\;x\;10^{-3}N/mm^{3},$ $6\;x\;10^{-3}N/mm^{3}$ , and $1.02\;x\;10^{-2}N/mm^{3}$ (average R2 equal to 0.95 ) respectively.

The goal of the experiment was to compare the reference profiles of the three samples with the profiles rendered by the proposed grasper. Let us assume that the reference and actual nonlinear stiffness to be $K_{d}$ and $K_{a}$ , respectively. While transmitting the reference stiffness profile from motor (M2) to end-effector, the nonlinearities associated with the 4-bar mechanism and passive components are also considered. Also, the gears were designed to provide the involute profile and avoid tooth interference to prevent the cogging and noise forces induced by the transmission. A proportional-integral (PI) controller was implemented to reduce the error between the reference and the actual stiffness coefficients. The high-fidelity stiffness profiles are rendered by considering the proportional ( P ) and integral ( I ) constants as 3.2 and 1,500. During this evaluation, the participant grasped the device to feel the stiffness profile. The rendered profiles by the haptic grasper are shown in Figure 8A. It is to be noted that the force values shown in these profiles are measured by a calibrated piezo sensor (vibro-actuator) that is positioned at the end-effector. The average root mean square error (RMSE) between the reference and rendered stiffness is $7.2\;x\;10^{-4}N/mm^{3}$ .

### Shape rendering

To analyze the shape rendering capability of the device, a reference sinusoidal is provided, and the tracking performance of the end-effector (grasper) is assessed. When the user moves their fingers in the grasping direction (along the *y*-axis), the two modular segments move in and out, providing the global shape profile (bumps and holes). The corresponding grasper’s motion is externally measured using a camera capture system. The rendered trajectory compared with the reference trajectory is shown in Figure 8B-(i). The RMSE and standard deviation are 0.21 and 0.11 mm, respectively. Also, the position tracking in the time domain is depicted in Figure 8B-(ii) to understand the dynamic behaviour. The computed tracking latency was up to 120 ms. The variations in the shape of segments having flattened surfaces, edges, active components, and so on can be provided to the user, as shown in Figure 7B. Since the device is equipped with two semi-circular modular segments, the shape profiles rendered by the grasper can only be curved shapes (sinusoidal functions).

### Texture rendering

**FIGURE 9:**
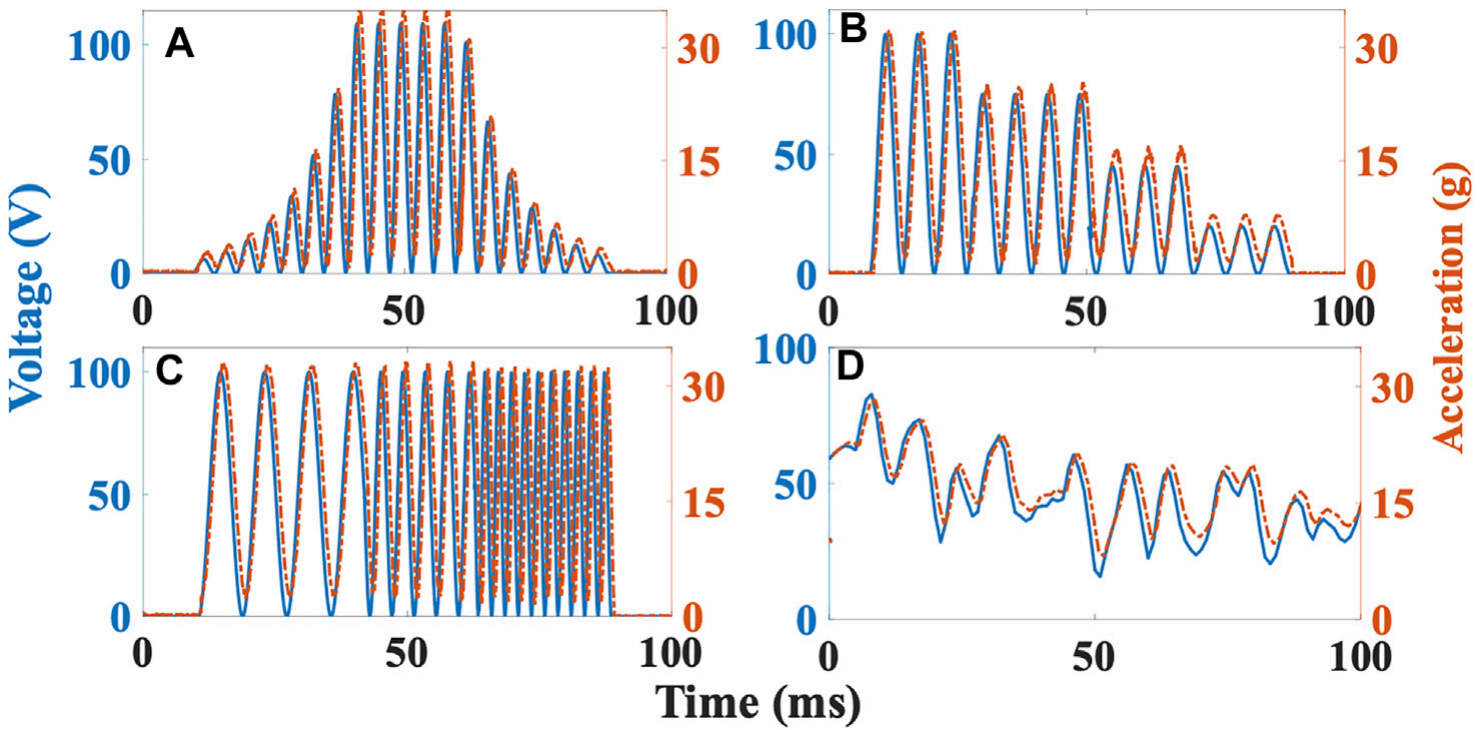
Acceleration responses corresponding to input voltage profile of vibro-actuator with load 20 g. **(A)** Variation of envelope (ramp up and ramp down) **(B)** Variation of amplitude **(C)** Variation of frequency **(D)** Random signal having all the three variations.

The goal of these experiments was to analyze the device’s texture rendering capability. Variations of textures are rendered by combining physical texture and the vibro-actuator. An experimental setup is built to examine the efficiency with which the vibro-actuator’s response is sensed by an accelerometer (ADXL357). One side of the actuator is attached to a fixed plate, and the other side is loaded with 20 g with a thin adhesive tape. The accelerometer is affixed above the load, and the vibration intensity of the movable mass is measured. The input voltage is varied from 0 to 120 V in four different patterns, and the corresponding accelerations are measured. The voltage is provided in terms of variations in the envelope, amplitude, frequency, duration, and the combination of all parameters (random signal). Figure 9 shows various levels of these parameters, i.e., two levels of the envelope (ramp up and ramp down) and four levels of amplitude and frequency. The change in each parameter relates to the textural variations distinctly. For instance, the changes in envelope and amplitude provide gradual/sudden bumps, grooves, and sharpness in textures. Similarly, the frequency changes render smooth/rough (high/low frequency) textures, and the duration represents the length of the textural surface. The corresponding output accelerations for an interval of 100 ms are shown in Figure 9. The generated maximum accelerations and displacements range from 0 to 50.2 g and 0–130 μm, respectively. Also, the average time delay between the voltage and acceleration signals is 5.7 ms. The results illustrate that the actuator’s accelerations and variations in vibrational characteristics generate multiple texture surfaces. Also, any vibrational response pattern can be accurately rendered because of the close resemblance between the voltage-acceleration signals. The accelerations rendered by the actuator are superior to the existing voice-coil actuators and other vibrotactile actuators (Koo et al., 2020). However, as the load increases, the acceleration rendered by the actuator decreases. Also, the applied forces in the rotary direction are sensed by an inbuilt current sensor in the motor. Based on these forces, the grasper’s position is altered, thus can render the shear feedback.

In Summary, the parameters affecting the transparency, such as backlash, friction, cogging effects and lack of realistic tactile feedback, have been addressed to provide transparent haptic sensations. A backlash compensation mechanism is proposed to eliminate backlash, and great care has been taken while designing the device to eliminate other effects like friction and cogging by choosing an involute profile for gear teeth. Also, the minimum no. of teeth for the pinion gear of the motors is kept above 17 to prevent tooth interference. For realistic tactile feedback, physical textures have been attached. The number of realizable textures are increased by using vibro-actuators underneath those textures.

## Psychophysical experiments

Three experiments were conducted to evaluate the participants’ perception of haptic sensations such as stiffness, shape, and texture rendered by the grasper. In addition to investigating the sensation discriminating abilities of the haptic grasper, these experiments were used to verify the efficacy of generating unified kinesthetic and tactile feedback. Each of the experiments was performed six times. The experimental setup shown in Figure 1 was utilized for performing the experiments. Ten participants (8 males, two females, aged: 22–30, all right-handed) have participated in the experiments after giving their consent. The participants were blindfolded throughout the experiments. There were no deficiencies in the perception abilities of any participants. Also, there was no time restriction imposed on the participants; hence they were permitted to touch the samples any number of times till they found a match. Moreover, if they did not find a correct match, they were allowed to report the same. The experimental procedure was approved by the IIT Madras Institutional Human Ethics Committee. The data analysis was done using the analysis of variance (ANOVA) method, performed using MATLAB software statistical tools. The significance level ( -value) for our study was chosen to be 0.05.

### Experiment #1: Stiffness discrimination task

In this experiment, two test blocks were considered: stiffness discrimination with and without texture (tactile feedback). Three cylindrical-shaped silicone specimens (similar to *Force rendering)* with the same length (3 cm) and radius (6 cm) but having different stiffness were chosen for analysis. The participants were asked to feel the stiffness profiles by probing the reference specimens along the *y*-axis using their index and thumb fingers and were sequenced based on the stiffness. Then they were allowed to interact with the haptic grasper similar to the specimen. The haptic grasper rendered three stiffness stimuli in the first block without any textures attached to the modular segment. In the second block, the textural surface similar to silicone specimens was affixed to the texture loading arm, and the grasper rendered the three stimuli (the methodology for generating stiffness through the device was explained in the previous section). The specimens and haptic grasper stimuli were given randomly. After each iteration, the participants reported the matching specimen corresponding to the stiffness stimuli rendered through the haptic device. These two blocks of the experiment illustrate the device’s stiffness rendering capability and the influence of texture on the stiffness modality.

The confusion matrices that describe the rendered versus actual stiffness without and with texture are shown in Table 1. The average cognitive accuracy for stiffness with and without texture is 95 ± 1.7% (mean ± SD) and 82.2 ± 2.54%, respectively. Statistical analysis with one-way ANOVA was performed between these two blocks to determine whether there was a significant difference between stiffness with and without texture. The results reveal that adding texture sensation had a significant effect on the stiffness rendering (F (1,28) = 33.81, p<0.001) .

Some other details related to statistical analysis are provided in the Supplementary Material.

**TABLE 1:** Confusion matrix for stiffness discrimination. (A) stiffness without texture. (B) stiffness with texture.

	E20	E30	E50	Relative accuracy (%)
E20	51	5	4	85.0
E30	5	48	7	80.0
E50	4	7	49	81.7

	E20	E30	E50	Relative accuracy (%)
E20	58	2	0	96.7
E30	1	56	3	93.3
E50	1	2	57	95.0

### Experiment #2: Shape discrimination task

In this experiment, the shape discrimination ability of the participants through the haptic grasper was examined. Initially, three cylindrical-shaped silicone samples with the same length (3 cm) and radius (6 cm) are chosen for analysis. Then, each specimen was moulded to form either a bump/hole at the centre with a width of 2 cm. The bump/hole was uniformly placed on the surface. During the experiment, participants were asked to feel the reference shape profiles of the three silicone samples by traversing forward and backwards the surface using their index and thumb fingers. Then they were allowed to experience the stimuli rendered by the haptic grasper by traversing it. When the user interacts with the grasper, the segment rotates and moves laterally about its midpoint. This motion depends on the position of the contact of the fingers with the haptic grasper. The specimens and haptic grasper stimuli were given randomly. After each trial, the participants reported matching specimens corresponding to the shape (bump/hole) stimuli rendered through the haptic device.

The confusion matrix that describes the rendered versus actual shape is shown in Table 2. The average cognitive accuracy for shape rendering is 92.8 ± 2.3%.

**TABLE 2:** Confusion matrix for shape discrimination.

	Bumps	Holes	Relative accuracy (%)
Bumps	85	5	94.4
Holes	8	82	91.1

### Experiment #3: Texture discrimination task

In this experiment, two test blocks, namely texture discrimination with and without stiffness, were considered. Four different signals illustrated in *Force rendering* were chosen for analysis. Each signal represents one property of the texture. The change in vibrational characteristics, namely amplitude, frequency, envelope, and duration, produce various surfaces such as gradual bumps/holes (VC1), sudden bumps/holes (VC2), smooth/rough surface (VC3), and length of the random texture, respectively. The participants were asked to feel these reference textural sensations using the index and thumb fingers. Sandpapers of different grits (roughness) were attached as physical textures to the texture loading arm. The sandpapers of grit sizes 40, 80, 120, and 240 with a thickness of 1 mm were utilized for analysis. The sandpapers’ dynamic attributes (roughness, depth, and size) are determined using the vibrational sensor for various inputs. So, to recreate reference surfaces, the properties of the attached sandpapers are varied through vibro-actuators by varying voltage inputs. In the first block, the participant was provided with a reference physical texture and the texture rendered by the haptic grasper without probing along the *y*-axis. In the second block, the participant was allowed to probe the grasper along the *y*-axis to feel both stiffness and texture. The stimulations of these textures were provided randomly. The signal patterns were repeated based on the participant requirement with a time delay of 300 ms to realize the sensation properly. These two blocks illustrate the device’s texture rendering capability and the influence of stiffness on the texture modality.

The confusion matrices that describe the rendered versus actual textural surfaces without and with stiffness are shown in Table 3. The average cognitive accuracy for textures with and without stiffness was 89.6 ± 3.7% and 84.2 ± 2.9%, respectively. The one-way ANOVA was performed between these two blocks. The results reveal that adding stiffness sensation had a certain effect on the texture rendering (F (1,28) = 5.77, p=0.023 ).

**TABLE 3:** Confusion matrix for texture discrimination. (A) Texture without stiffness. (B) Texture with stiffness.

	VC1	VC2	VC3	VC4	Relative accuracy (%)
VC1	49	4	5	2	81.7
VC2	3	50	4	3	83.3
VC3	6	2	50	2	83.3
VC4	2	4	1	53	88.3

	VC1	VC2	VC3	VC4	Relative accuracy (%)
VC1	51	2	5	2	85.0
VC2	2	55	1	2	91.7
VC3	5	2	53	0	88.3
VC4	2	1	1	56	93.3

## Results and discussions

In this section, the outcomes related to psychophysical experiments are discussed in detail. In the stiffness, shape, and texture discrimination tasks, the participants could easily discriminate between different stiffness (nonlinear), shape and texture profiles and match the reference stimulus provided by the haptic grasper.

To understand the influence of stiffness modality on texture modality and vice-versa, the average cognitive accuracy of both experiments is normalized and considered for analysis. In the stiffness discrimination experiment, for the applied user’s force, the deformation of the haptic grasper is more in the case of the E20 specimen because of its low stiffness, and therefore, the users could easily identify this specimen. Based on the stiffness constant, the deformation of the E30 specimen is higher than the E50 specimen for the same applied force. The participants should have easily differentiated between E30 and E50 specimens. However, the users became confused between these two specimens because of their low deformation compared to the E20 specimen. The relative accuracy of the E50 specimen is higher than the E30 specimen since it has high stiffness. From the experimental analysis, it is understood that the user could accurately recognise the softest (E20) specimen, followed by the hardest (E50) specimen and then the E30 specimen. Although the accuracy of identifying specimens has increased by adding the texture modality, the order remains the same regardless of adding other modalities. In the texture discrimination experiment, the user could easily identify the length of random texture (VC4) because of the variations in both amplitude and frequency compared to other profiles. The sudden change of four levels of variations in amplitude (VC2) and frequency (VC3) could also be recognised by the users next to VC4. The gradual amplitude variations (VC1) could not be recognised because the user could not identify the slight change in amplitude and had been confused with other profiles. However, when the stiffness modality is added to the texture, the variations in amplitude (VC2) are sharply felt by the user with the applied force, thus taking the superior position compared to VC3. The other order remains the same with added stiffness modality.

**FIGURE 10:**
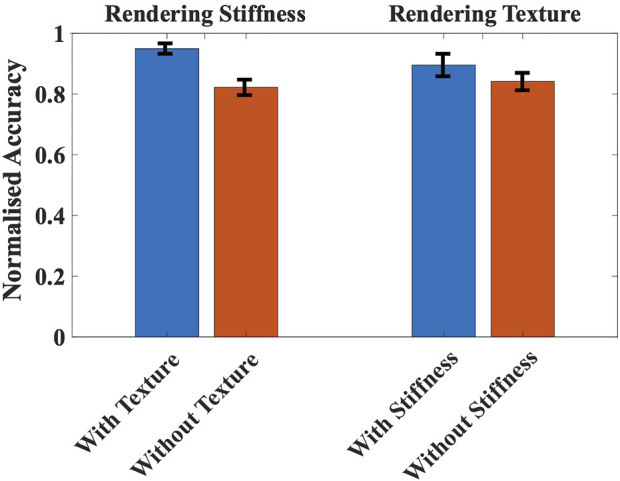
Comparison of normalized mean accuracy of rendering stiffness with/without textures and rendering texture with/without stiffness. The error bars indicate the standard deviation of the normalized accuracy.

The metrics of interest in these experiments were relative cognitive accuracy and the standard deviation. The collected data of the two groups passed both the Shapiro-Wilk normality test and Levene’s variance homogeneity test. The stiffness/texture discrimination experiments show the normalized mean accuracy of stiffness with/without textures (Case A) and rendering of texture with/without stiffness (Case B) along with their error bars is shown in Figure 10. In Case A, the cognitive accuracy of the combination of stiffness and texture is 13.5% better than stiffness alone. The accuracy of feeling the kinesthetic cues (stiffness and shape) would increase because of integrating the tactile cues (textures) into the interaction. In Case B, the combination is 6% better than the texture alone. Therefore, in any case, the participants could feel the sensations naturally when unified sensations are provided as haptic feedback. Also, the one-way ANOVA and posthoc analysis (Tukey HSD test) were performed between the “stiffness with texture” and “texture with stiffness” blocks to determine the influence of each modality on the haptic feedback. The results show that stiffness rendering with texture would significantly affect haptic feedback than texture rendering with stiffness [F (1,28) = 21.14, p<0.001 ], with a mean difference of 5.4. Comparing the results between rendering texture and stiffness individually, the texture comprising variations both in amplitude and frequency would more significantly impact the user’s perception, followed by low stiffness environments. However, when each modality is combined with another, the stiffness cues with textures are identified more accurately than the texture cues with stiffness because when the user applies force on the environment, the stiffness modality dominates texture modality. Therefore, the performance of stiffness with textural feedback is significant compared to texture with stiffness feedback. Also, the shape discrimination experiment results demonstrate that the participants could recognize similar shapes sensitively using the haptic grasper. Compared to some works in literature rendering the shape modality (Sun et al., 2018; Whitmire et al., 2018), the proposed haptic grasper moving rotationally and longitudinally aids in matching the exploration trajectory with the shape profile of the virtual environment. The grasper further augments the fidelity of the haptic feedback by concurrently providing the unified feedback of stiffness, shape, and texture to multiple fingers. This concurrent rendering of multiple modalities enhances the user’s sensitivity to recognize the individual modality. Additionally, from the system characterization and the psychophysical experimental results, the textural feedback rendered through the combination of physical texture and vibro-actuators is naturally felt by the users. However, the reason for lower performance (user studies and questionnaire) is the incapability of representing a large variant of the attached physical texture.

Additionally, the proposed haptic grasper workspace is limited, as mentioned in the device specifications (Table 4), because the implementation is a proof of concept for demonstrating the idea of using the textural surfaces backed by vibro-actuators along with stiffness modality to improve the haptic fidelity. However, the design can be adapted to a smaller/lighter form factor which can be linked to a standard serial/parallel kinematic arm for extending the workspace. Another key challenge of this design is the miniaturization of the various mechanisms, which can be addressed by accessing precision manufacturing resources.

**TABLE 4:** Specifications of the proposed haptic grasper.

Specification	Proposed haptic grasper
Length of the virtual object rendered	65 mm
Maximum deformation rendered	12.4 mm
Angular range of motion for each grasper	180^o^
Angular range of motion for each texture	89^o^
Input/Output DoF	3-DoF

In comparison with the state of literature (Najdovski et al., 2014; Benko et al., 2016; Choi et al., 2016; Whitmire et al., 2018), the performance of the proposed haptic grasper is improved in terms of providing the combination of kinesthetic and tactile modalities simultaneously, and accuracy of rendered haptic modalities such as shape, texture and stiffness.

### Virtual reality demonstration and applications

**FIGURE 11:**
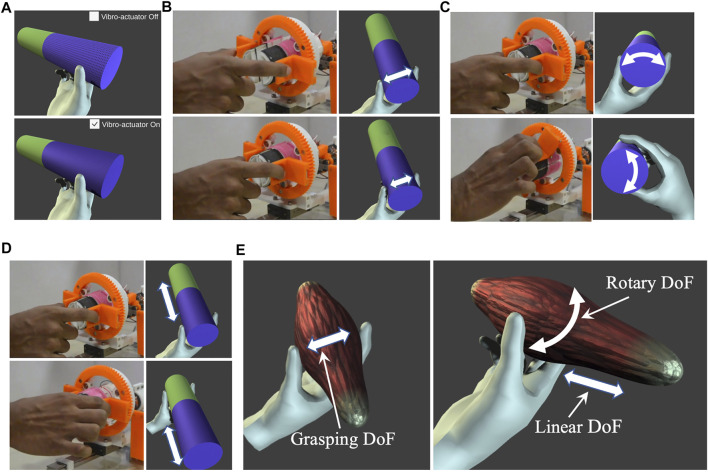
A cylindrical virtual object is created and rendered to the user to highlight the attributes of the proposed haptic grasper. **(A)** Textural feedback with/without Vibro-actuator. Three degrees of freedom (DoF) of the cylindrical object are provided to the user. **(B)** Grasping DoF **(C)** Rotary DoF **(D)** Linear DoF. **(E)** A telesurgical application demo is also rendered to the user allowing him to explore the virtual muscle characteristics such as texture, shape, and stiffness. The double-headed arrows represent the grasper’s direction of motion.

To demonstrate the virtual reality (VR) interaction with various environments, two VR scenarios are custom-developed in Unity 3D to highlight the attributes of the haptic grasper. The 3-DoF of the haptic device is synchronized with LabVIEW software, and these signals are sent to the virtual environments made in Unity 3D through the User Datagram Protocol (UDP) channel. Ten participants were immersed in these experiments to evaluate the naturalness of these interactions, holding the device with their fingers. The first application emphasizes various abilities of the device, such as the degrees of freedom, stiffness, shape, and texture modalities. In this scene, a cylindrical object is chosen with two different textures (green and violet), as shown in Figure 11. The vibro-actuators placed underneath the textures help realize several other textures by changing the vibration characteristics. Hence, the vibro-actuators enable the user to experience a greater number of textures than the textures physically present in the device. The status of the vibro-actuator, whether it is ON or OFF, is also shown in the virtual environment. When the vibro-actuator is ON, the various textures that the user experiences are accordingly modified in the virtual scenario. The user can move his hand forward and backward (linear DoF), rotate (rotary DoF), and move laterally (grasping DoF). When the user applies the force on the device, the cylinder in the virtual environment deforms, and thus, stiffness modality is realized. Similarly, the shape modality is rendered when the user moves his hands longitudinally or rotates. However, since the segments are modular, physical segments with appropriate shapes (prismatic shape) can also be used to render geometries with abrupt features such as sharp edges or corners, active components and so on.

The second VR scenario is related to the application of telesurgery, exploring various features of a virtual muscle. In this demo, the virtual muscle characteristics (haptic feedback) are rendered to the participants users by mapping with the device workspace (as mentioned in Table 4), as shown in Figure 11E. The user could feel the shape and texture of the virtual muscle and palpate it (stiffness modality) to assess its healthiness. When the user applies lateral forces, the output torque of the drive motor is controlled accordingly to render the stiffness of the virtual environment (muscle), which is explained in *Force rendering*. The shape information of the muscle is experienced by the user’s longitudinal and rotary movement of the haptic grasper, which is explained in *Shape rendering*. The shape and stiffness of the muscle are rendered simultaneously by utilizing the coordinated movements of all the three DoF of the device. The users experiencing these muscle features could differentiate between a healthy and an affected one. The users could also identify the precise position of the affected muscle area and the differentiating factor with respect to the healthy muscle in terms of change in shape or stiffness. Participants’ responses were recorded using a questionnaire based on a Likert seven-point scale (1-Strongly disagree, 7-Strongly agree) to assess the user’s experience of different haptic sensations in virtual environments. The questions were presented to the participants in random order. The mean and standard deviation of the scores was calculated based on the user’s rating, and the same has been reported in Table 5. All the participants found that the device was easy to use, and the haptic sensations rendered were intuitive and realistic. Moreover, most participants were excited and stated that they felt as if they were interacting with the natural environment and remarked that the virtual experience enhanced the rendered haptic feedback. At the same time, a couple of participants suggested improving the naturality of textures generated through vibro-actuation but were delighted with this experience compared to existing haptic/vibrotactile controllers (Ohka et al., 2005; Sun et al., 2018; Whitmire et al., 2018).

**TABLE 5:** Questionnaire for User Evaluation using Likert seven-point scale.

	Questions	Mean	SD
Q1	It was comfortable and easy to use the haptic grasper	6.5	0.3
Q2	It was easy to discriminate the stiffness rendered through the device	6.3	0.5
Q3	The stimuli generated by the device allow feeling the shape sensations	5.9	0.6
Q4	I experienced tiredness after the experiments	1.2	0.4
Q5	The vibrations from the actuator are overpowering the texture sensations	1.8	1.2
Q6	Distinct textural properties are felt naturally	5.8	0.7
Q7	The combination of kinesthetic and tactile feedback provided realism to the interaction	6.8	0.2
Q8	The device could not represent the reference (virtual/remote) object transparently	1.3	0.5
Q9	The haptic sensations provided by the grasper are abnormal	1.5	0.4
Q10	The modularity of the device aids in representing a variety of environments	6.2	0.2
Q11	The experience with virtual environments is consistent with the real-world experience	6.1	0.3

The proposed device finds its use in many human-machine interface applications such as telesurgical systems, training medical apprentices, space and underwater exploration, rehabilitation, industrial operations, and entertainment (Kawasaki, 2015; Enayati et al., 2016). For example, in telesurgical applications, haptic feedback in the form of stiffness and texture sensations would assist the surgeon in feeling the characteristics (healthiness) of tissues/muscles while palpating, which has been demonstrated to the participants. Furthermore, sensing the tissue properties helps surgeons identify medical issues varying from a lumbar puncture to tumor detection (Enayati et al., 2016). Also, because of variations in textural behavior in the initial phase of the disease, providing textural feedback along with stiffness is essential. Similarly, the sensations provided by the device could be used to upskill medical apprentices and could be of use in space and underwater exploration scenarios to get a feel of remote objects’ properties.

Additionally, the modularity of the device extends its usage for a variety of remote/virtual environments. The drive motors are mounted directly in the device to keep design complexity minimum for the initial prototype. In future versions, the motors can be placed remotely by using Bowden cable transmission to enhance the workspace and reduce the device’s weight.

## Conclusion and future scope

This paper presents a graspable haptic interface that enables users to grasp and interact with different virtual/remote environments with haptic modalities, such as stiffness, shape, and texture. Both kinesthetic and tactile feedback are presented simultaneously in grasping, linear, and rotary DoF. The system characteristics and performance of the device have been elaborated. The psychophysical experiments and questionnaire using Likert seven-point scale results demonstrate that the haptic grasper could provide the natural feel of interacting with various virtual objects/environments. Furthermore, the analysis relating to the performance of the discrimination tasks for stiffness, shape, and textural feedback has been studied using statistical analysis. It is also found that textural feedback plays a prominent role in rendering the stiffness. Finally, applications of the haptic grasper have been reported with a detailed explanation of the use of the device for telesurgical systems. Future works comprise miniaturization and extending the haptic grasper to a wearable device, thus enhancing the workspace and applications.

## Data Availability

The original contributions presented in the study are included in the article/Supplementary Material, further inquiries can be directed to the corresponding author.
