# Supplementary material for: Design and realization of a novel haptic graspable interface for augmenting touch sensations
Source: Front Robot AI. 2022 Sep 28;9:927660. doi: 10.3389/frobt.2022.927660 (PMC9554353; doi:10.3389/frobt.2022.927660)
Supplement: Supplementary file 1 [file DataSheet1.PDF]

Supplemental document

1. To understand the working of the proposed haptic grasper better, the following figures are provided. Figure 1 elaborates the various DoF's of the device. Figure 2 elaborates the preloading of vibro-actuator.

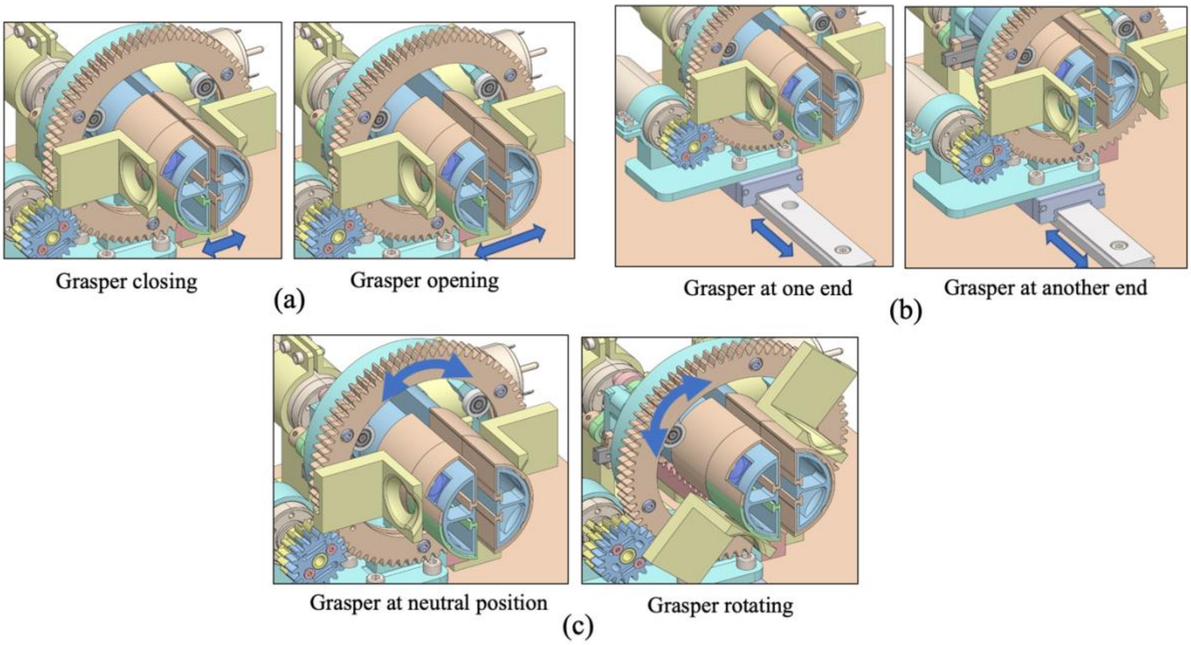

Figure 1: Three degrees of freedom (DoF) of the Haptic Grasper are (a) Grasping DoF (b) Linear DoF (c) Rotary DoF. The double headed arrows in each of the models show the direction of motion of the grasper. The modular segments are attached with texture loading arms and vibro-actuators, which are fixed to four-bar mechanism and can only move laterally.

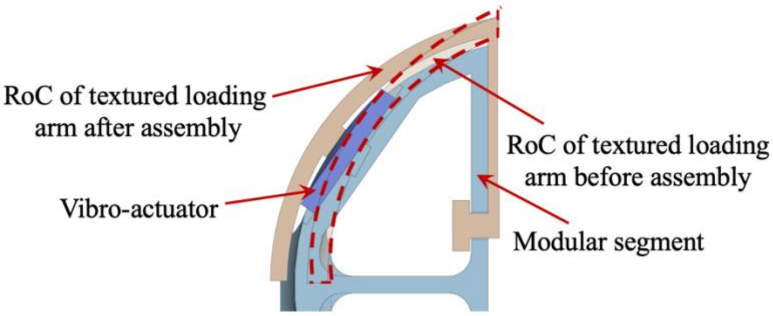

Figure 2: Vibro-actuator preloading. The textural arm is 3D printed with a smaller radius of curvature (RoC) and after the assembly, the RoC increases to accommodate the vibro-actuator leading to its preloading

2. Some of the details related to statistical analysis are provided below:

The metrics of interest in the experiments were relative cognitive accuracy and the standard deviation. The collected data of the groups have passed both the Shapiro-Wilk normality test and Levene's variance homogeneity test. The Shapiro-Wilk test is tested at $\alpha = 0.001$ level of significance, and since $p > 0.001$ for all the groups, the conclusion is that all the groups are

normally distributed. For Levene's test, α is considered 0.001, and since $p > 0.001$, all the groups satisfy the condition of homogeneity of variance. So, the test of ANOVA (parametric) is considered for analysis. A post hoc analysis (Tukey HSD test) is performed to identify which group is significantly different from the other specifically. It is concluded that the 'stiffness with texture' and 'texture with stiffness' are significantly different, with a mean difference of 5.4 and p value less than 0.001.
